# Structural Deficits in the Frontotemporal Network Associated With Psychopathic Traits in Violent Offenders With Schizophrenia

**DOI:** 10.3389/fpsyt.2022.846838

**Published:** 2022-04-12

**Authors:** Ningzhi Gou, Juntao Lu, Simei Zhang, Xiaoxi Liang, Huijuan Guo, Qiaoling Sun, Jiansong Zhou, Xiaoping Wang

**Affiliations:** ^1^Department of Psychiatry, National Clinical Research Center for Mental Disorders, The Second Xiangya Hospital of Central South University, Changsha, China; ^2^Shenzhen Mental Health Center, Shenzhen Kangning Hospital, Shenzhen, China

**Keywords:** schizophrenia, violence, gray matter volume, psychopathic traits, frontotemporal network

## Abstract

People with schizophrenia (SZ) are at increased risk of violence compared to the general population. However, the neural mechanisms of violent behavior in patients with SZ are still unclear due to the heterogeneity of the diseased population. In this study, we aimed to examine the neural correlates of violent behavior in SZ and to determine whether the structural deficits were related to psychopathic traits. A total of 113 participants, including 31 SZ patients with violent behavior (vSZ), 39 SZ patients without violent behavior (nvSZ), and 43 healthy controls (HC), completed the T1-weighted magnetic resonance imaging (MRI) scan and were analyzed using voxel-based morphometry approach. The psychopathic traits were assessed using the Psychopathy Checklist: Screening Version (PCL:SV). The results showed decreased gray matter volume (GMV) in the vSZ group in the right temporal lobe and bilateral inferior frontal gyri compared to HCs; while reduced GMV in the inferior parietal lobe, parahippocampal and orbital frontal gyri was found in the nvSZ group compared with HCs. Correlation analyses showed that psychopathic traits were negatively associated with the GMV in the right superior temporal and left fusiform gyri in the vSZ group, indicating that psychopathic traits, as reflected by the score of antisocial factor, might be related to structural deficits in the temporal lobe, which led to a propensity to violent behavior in patients with SZ. Our findings suggest that violent behavior in patients with SZ might have a personality background associated with the frontotemporal network aberrance. In future studies, we need to take a closer look at psychopathic traits for better understanding of the mechanism of interpersonal violence in patients with SZ and to explore whether the imaging findings from this study can serve as a biomarker to predict future violent behaviors and community living.

## Introduction

Patients with schizophrenia (SZ) are at increased risk of engaging in violence toward others (1, 2) and account for around 10% of all homicide offenders (3, 4). A 38-year total population study in Sweden showed that 10.7% of men with SZ and related non-affective psychoses were convicted of violent offenses within 5 years from their initial diagnosis (5). Given that violence is a multifaceted phenomenon (6, 7), the neurobiological basis of violent offenses in patients with SZ is still unknown. Thus, the exploration of neurobiological underpinnings of violence in patients with SZ can help inform the biological evaluation of the risk for violence as well as develop individualized disease prediction and intervention for violent behavior.

Numerous studies have reported abnormal morphological changes in violent offenders with schizophrenia (vSZ), which involved the frontal and temporal lobes, as well as subcortical nuclei (8–10) compared to non-violent patients with schizophrenia (nvSZ). For instance, studies suggested that reduced gray matter volume (GMV) in the frontal lobe, temporal lobe (10–12), and hippocampus (13–15) were associated with violent behavior in patients with SZ. Despite the above discoveries, inconsistent results were apparent. A crucial region belonging to the limbic system, the amygdala, has been involved in controversial results (9, 16), as some studies reported increased volume of the amygdala in vSZ relative to nvSZ (13, 17, 18), while others reported opposite results (19, 20). Another region exhibiting controversial findings was the orbitofrontal cortex. For instance, Hoptman et al. found that larger gray and white matter volumes were associated with higher levels of aggression in patients with SZ (21). Furthermore, a few neuroimaging studies reported no significant differences in the brain structure between vSZ and nvSZ (17, 22–24). The mixed findings might be derived from the heterogeneous sample characteristics and various definitions of violence (20, 25, 26). Regarding these issues, more study exploring the intrinsic brain structure of violence in patients with SZ is needed.

Reasons for violent behavior in SZ patients can be classified into intrinsic and external factors, such as young age, deviant personality, childhood trauma, and substance abuse (27), which may contribute to neurobiological variability of violence in SZ. An explicit viewpoint is that those domains that exist in different populations might be associated with the pathology of violence (28), suggesting that intrinsic factors, such as the deviant personality traits, were more reflective of the neurodevelopmental basis of violence. A series of previous epidemiological studies have recognized that psychopathy, which referred to a personality type characterized by emotional callousness, lack of empathy, grandiose interpersonal style, impulsivity, and persistent antisocial behaviors (29), was significantly associated with violence in patients with SZ (28, 30). For instance, Fullam et al. found that 20–30% of patients with SZ were comorbid with psychopathy in forensic psychiatric settings (31, 32). In such a context, patients with SZ who have psychopathic traits might be at higher risks for violence; thus, the exploration of the anatomical basis of psychopathic traits may help reveal the neurological basis of violence in the population with SZ.

Literatures have revealed that the anatomical basis of psychopathic traits might be more specifically associated with regions involved in emotional processing and integration of emotion into cognition, including the limbic and paralimbic regions (33–35), it supports the finding that high levels of psychopathic traits might be resulted from the dysfunction of the frontotemporal-limbic network (36). However, relevant studies examining the neural basis of psychopathic traits in violent patients with SZ are rare, especially on a brain morphological level. A previous study on white matter (WM) integrity showed extensive reduction of WM in vSZ, which was positively associated with the level of psychopathic traits (37); another functional MRI study revealed a blunted amygdala response to fearful faces in male vSZ who had high levels of psychopathic traits (32). Some other comparative studies examining the differences in brain structure between vSZ and those patients with ASPD/psychopathy found common structural changes in these populations involving the hippocampus (13, 15), anterior cingulate cortex (22), and sensorimotor cortices (38). The previous findings demonstrated that the structural profiles underlying violent offenses in psychopathic individuals and vSZ were partly overlapped and dominantly involved in emotion processing and high-order cognition.

Based on the above evidence, we hypothesized that the vSZ might have structural deficits in the frontotemporal-limbic network as compared to nvSZ; furthermore, the network abnormalities might be associated with a higher level of psychopathic traits, which lead to a propensity to violence in SZ. The aim of the present study was to investigate the neural correlates of violence in patients with SZ and their clinical characteristics, and to explore the association between the neural correlates and psychopathic traits in the vSZ group. A whole brain GMV analysis derived from the voxel-based morphology (VBM) was conducted between violent patients, non-violent patients and healthy control groups (vSZ vs. nvSZ, vSZ vs. HCs, and nvSZ vs. HCs), which was followed by a correlation analysis to further reveal whether these structural deficits were associated with high levels of psychopathic traits. Hopefully, this study would shed light on the mechanisms of violence in SZ and further facilitate early intervention against violence in patients with SZ.

## Materials and Methods

### Participants

A total of 31 violent offenders with SZ were recruited from November 2011 to November 2021 from the forensic psychiatric department of the Second Xiangya Hospital of Central South University. In this study, violence was defined as having committed a homicide or other assaults that caused severe physical injury to others. Thirty-nine age-matched control patients, who had never been engaged in violence, were recruited from general inpatients or outpatients of the psychiatry department of the same hospital. All the patients met the inclusion criteria: (1) male; (2) aged between 18 and 50 years; (3) with an IQ higher than 70; and (4) meeting the diagnosis of schizophrenia based on a semi-structured interview corresponding to the International Classification of Diseases Version 10 (ICD-10). Patients meeting the following criteria were excluded: (1) with a history of alcohol or substance abuse; (2) with a history of major neurological disorders or unstable medical conditions; (3) with a history of head trauma (loss of consciousness for more than 5 min); and (4) with MRI contraindications. Forty-three age-matched healthy controls (HC) were recruited, the inclusion and exclusion criteria were the same as those for the patients, except that they and their first-degree relatives must not meet the ICD-10 criteria for any Axis-I psychiatric disorders. All participants provided written informed consent after fully informed of all the study components. The study was approved by the Ethics Board of the Second Xiangya Hospital.

### Socio-Demographic and Clinical Assessments

Socio-demographic and clinical information were collected through a self-designed standardized form, which included residence and marital status, duration of illness, medications, and the assessment of personality and psychotic symptoms.

The Brief Psychiatric Rating Scale (BPRS) (39) was used to estimate the overall psychotic symptoms. This scale included five dimensions, i.e., anxiety-depression, withdrawal, thought disturbance, activation, and hostility-suspicion, with the total score > 35 indicating an acute episode.

The Psychopathy Checklist: Screening Version (PCL:SV) (40, 41) was used to estimate personality traits. The scale has a 4-factor structure consisting of 12 items in total, with each item scored on a 3-point scale (0 = does not apply, 1 = partially applies, and 2 = definitely applies; the total score ranged from 0 to 24). The factors 1–4 reflected interpersonal deficit, emotional deficit, socially deviant lifestyle, and antisocial behavior, respectively. Trained research staff conducted in-depth semi-structured interviews with the patients and reviewed all the file records, including criminal and medical records.

### Neuroimaging Acquisition and Processing

Neuroimaging data were collected using a 3-tesla (3T) MRI scanner (Philips Medical Systems). High spatial resolution, 3-dimensional (3D) T1-weighted images were used for morphometric measures. The following parameters were used for the sequence: repetition time/echo time (TR/TE) = 8.2/3.8 ms, field of view (FOV) = 256 × 256 mm, matrix size = 256 × 256, voxel size = 1 × 1 × 1 mm, and 188 contiguous 1-mm sagittal slices. All images were processed and analyzed using the CAT12 toolbox (C. Gaser, Structural Brain Mapping Group, Jena University Hospital, Jena, Germany; http://dbm.neuro.uni-jena.de/cat/) in SPM12 (Wellcome Trust Centre for Neuroimaging, London, UK; https://www.fil.ion.ucl.ac.uk/spm/software/spm12/).

Data were analyzed with the following procedure: (1) registration of all images to the same template image; (2) normalization of the whole anatomic image to the same standard stereotactic space; (3) segmentation of the anatomical image with signal intensity and probable information to normalize customized template; (4) modulation for the intensity of the white matter images with the surrounding voxels compressed or expanded (modulation for non-linear effects only in case of relative changes corrected for total intracranial volume); and (5) smoothing with an 8 mm Gaussian Kernel for the group analysis. Data processing included a two-step quality assurance. Firstly, all images were visually inspected for artifacts (prior to pre-processing); secondly, all images underwent statistical quality control for inter-subject homogeneity and overall image quality using the CAT12 toolbox (“check homogeneity” function) after segmentation. This second step also included a visual inspection procedure for newly introduced artifacts.

### Statistical Analyses

Statistical analyses of demographic and clinical data were performed using the IBM SPSS Statistics 25 (Armonk, NY: IBM Corp.). Categorical variables were presented as frequency (percentage), and continuous variables were presented as mean ± standard deviation (SD). The normality of the continuous variables was tested using the Shapiro-Wilk test. For normally distributed data, parametric one-way analysis of variance (ANOVA) or two-sample *t*-test was used to analyze inter-group differences, and partial correlation was used to calculate the correlation between imaging and clinical data with age, education and duration of illness controlled. For non-normally distributed data, non-parametric Mann-Whitney U test was used to analyze inter-group differences, and spearman correlation was used to examine the correlation between imaging and clinical data. Categorical variables were analyzed using Chi-square test. The threshold of statistical significance was set at *P* = 0.05.

We performed statistical analysis on imaging data in the SPM12 statistical module. Differences in whole-brain GMV between the three groups were compared using voxel-wise one-way analysis of variance (ANOVA), with age, education and the total intracranial volume (TIV) controlled (*P* < 0.001, uncorrected). Clusters that survived in the ANCOVA analysis were saved as masks for further *post-hoc* pairwise *t*-test, the threshold was set at cluster-level *P* < 0.05 with family-wise error (FWE) corrected for multiple comparisons. To further verify our results, a permutation test with Threshold-Free Cluster Enhancement (TFCE) (42) for multiple comparison corrections was conducted, and *P*-values were calculated using permutation-based statistics (5,000 permutations), with FWE correction (*P* < 0.05); and then, pairwise comparisons were performed using the mask from the ANCOVA analysis (*P* < 0.05, FWE corrected).

## Results

### Demographic and Clinical Characteristics

The demographic and clinical characteristics of vSZ, nvSZ and HCs are summarized in Table 1. No significant difference was found in age [*F*_(2, 110)_ = 1.19, *P* = 0.31] between the three groups, and vSZ had a significantly lower level of education than the other two groups [*F*_(2, 110)_ = 6.00, *P* = 0.003]. As for marital status, a slight difference (χ^2^ = 12.85, *P* = 0.045) was found between the three groups, with more than 80% of the vSZ were single. With regard to medication use, more than half of the patients had used antipsychotics, but few of them was on regular medication. The types of drugs they used mainly included sulpiride, perphenazine, risperidone, quetiapine, and clozapine. The two groups of patients had similar duration of illness (*P* > 0.05).

**Table 1:** Characteristics of demographic and clinical data of all groups.

**Variables**	**vSZ (*n* = 31)**	**nvSZ (*n* = 39)**	**HC (*n* = 43)**	***F/χ^2^/T***	***P*-value**
Age (years) (mean ± SD)	30.64 (7.64)	29.23 (7.58)	31.77 (7.17)	1.189	0.308
Education (years) (mean ± SD)	9.97 (2.44)	11.95 (3.20)	12.23 (3.01)	6.000	**0.003**
≤ 6 (*n*, %)	2 (6.5)	1 (2.6)	2 (4.7)	8.184	0.085
≤ 12	27 (87.1)	35 (89.7)	30 (69.8)		
≥13	2 (6.5)	3 (7.7)	11 (25.6)		
**Marital status (** ***n*** **, %)**					
Single	26 (83.9)	27 (69.2)	24 (55.8)	12.851	0.045
Married	3 (9.7)	7 (17.9)	16 (37.2)		
Divorced	2 (6.5)	1 (2.6)	3 (7.0)		
**Residence (** ***n*** **, %)**					
Living alone	5 (16.1)	4 (10.3)	–	0.883	0.643
Living with family	21 (67.7)	26 (66.7)	–		
Others	5 (16.1)	9 (23.1)	–		
**Medications (** ***n*** **, %)**					
No	9 (29.0)	17 (43.6)	–	1.568	0.211
Yes	22 (71.0)	22 (56.4)	–		
Duration of illness (months)	73.26 (61.66)	54.33 (52.73)	–	1.384	0.171
BPRS total score	52.54 (9.43)	51.62 (7.31)	–	0.467	0.642
**PCL:SV**					
PCL:SV (total score)	5.77 (3.68)	1.97 (2.21)	–	4.365	**<0.001**
PCL:SV (factor 1)	0.35 (0.61)	0.38 (0.78)	–	−0.158	0.875
PCL:SV (factor 2)	1.81 (1.42)	0.47 (0.96)	–	4.219	**<0.001**
PCL:SV (factor 3)	2.58 (2.01)	0.94 (1.07)	–	3.504	**0.001**
PCL:SV (factor 4)	1.06 (1.15)	0.17 (0.46)	–	3.808	**<0.001**
TIV (cm^3^)	1,271.53	1,210.32	1,241.49	0.664	0.517
	(234.08)	(209.32)	(222.68)		

*vSZ, violent offenders with schizophrenia; nvSZ, non-violent patients with schizophrenia; BPRS, Brief Psychiatric Rating Scale; PCL:SV, Psychopathy Checklist: Screening Version; TIV, total intracranial volume*.

As for clinical characteristics, vSZ had significantly higher PCL:SV total score (*T* = 4.37, *P* < 0.001), as well as scores of factor 2 (*T* = 4.22, *P* < 0.001), factor 3 (*T* = 3.50, *P* = 0.001), and factor 4 (*T* = 3.81, *P* < 0.001), indicating that vSZ had significant higher levels of psychopathy than nvSZ. There was no significant difference on the level of psychotic symptoms measured with the BPRS total score between the vSZ and nvSZ groups (Table 1).

### VBM Analysis

**Figure 1:**
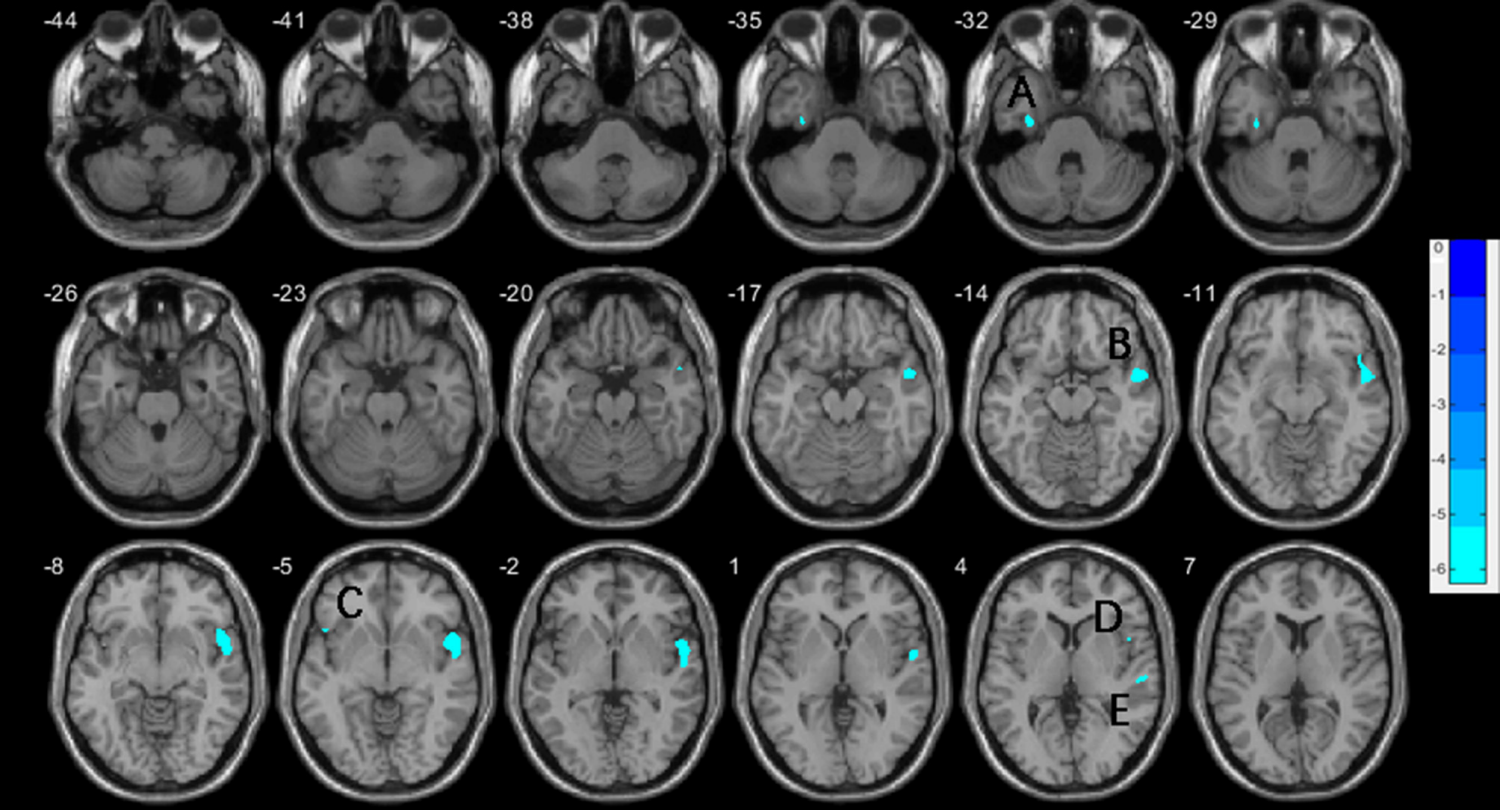
Decreases of gray matter volume in the vSZ group compared to HCs, with age, education, and TIV controlled. (A) left fusiform gyrus; (B) right superior temporal gyrus; (C) left inferior frontal gyrus, orbital; (D) right inferior frontal gyrus, operculum; (E) right superior temporal gyrus. TIV, total intracranial volume.

**Figure 2:**
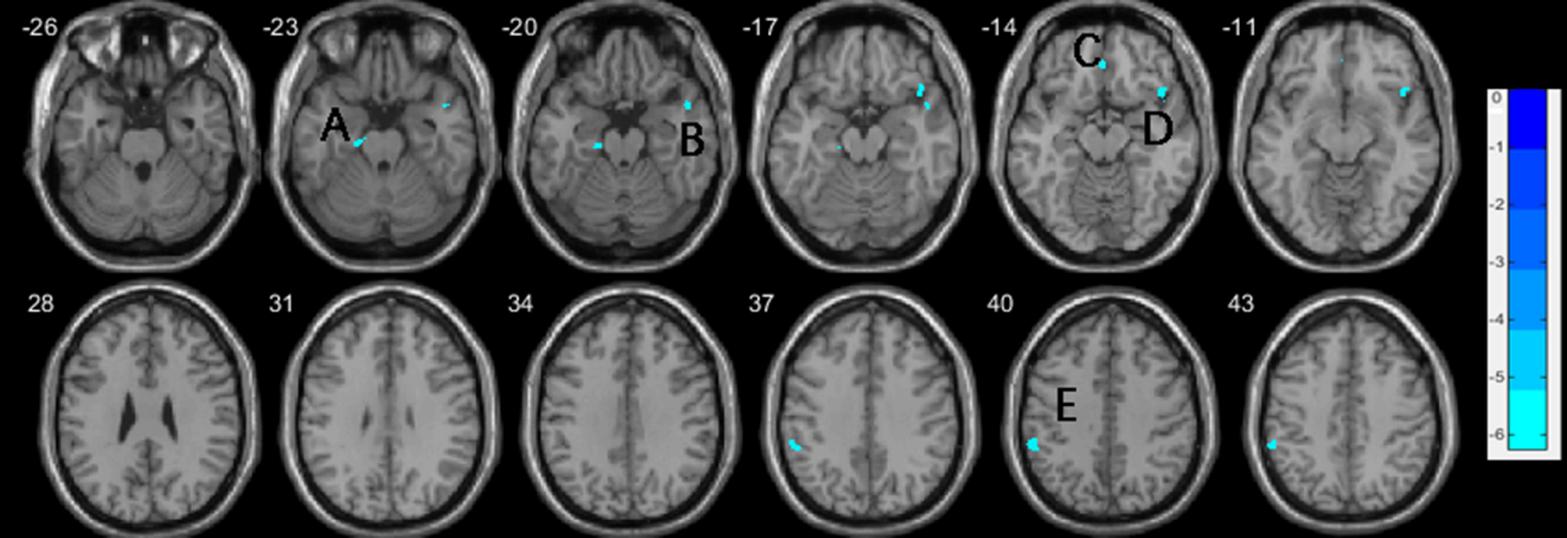
Decreases of gray matter volume in the nvSZ group compared to HCs, with age, education, and TIV controlled. (A) left parahippocam pal gyrus; (B) right temporal Pole: superior temporal gyrus; (C) right rectus, extending to bilateral medial orbital frontal gyrus; (D) right inferior frontal gyrus, orbital; (E) left inferior parietal lobe. TIV, total intracranial volume.

**Figure 3:**
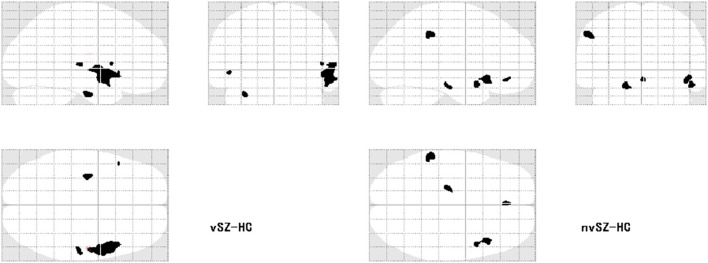
Regions showing reduced gray matter volume in the vSZ and nvSZ groups compared to HCs on the transparent brain.

One-way ANCOVA (*P* < 0.001, uncorrected) revealed significant differences in GMV in widespread regions, including the right superior temporal gyrus (STG), right insula gyrus (INS), right temporal pole (superior and middle temporal gyrus [TP]), left fusiform gyrus (FFG), left parahippocampal gyrus (PHG), bilateral medial orbital frontal gyri (mOFG), bilateral orbital inferior frontal gyri (IFGorb) and left inferior parietal lobe (IPL), between the three groups. *Post-hoc t*-test showed that, compared to HCs, vSZ showed significantly reduced GMV in a large cluster from the right STG to the insula (INS), TP, and IFGorb. Other regions showing reduced GMV in the vSZ group were the left FFG, IFGorb, and right opercular IFG (IFGoper) (*P* < 0.05, FWE corrected; Table 2; Figures 1, 3). In the nvSZ group, reduced GMV was found in the left IPL, PHG, the bilateral mOFG, and the right superior TP and IFGorb, as compared to the HC group (*P* < 0.05, FWE corrected; Table 2; Figures 2, 3). Marked decrease was found in the GMV of the right temporal lobe and bilateral IFG in the vSZ group rather than the nvSZ group, as compared with HCs. In addition, a more pronounced decline of GMV in the left IPL was found in the nvSZ group as compared to the vSZ group, although there was no significant difference. The results obtained by using TFCE were quite similar with our original results (see Supplementary Table 1; Supplementary Figures 1–3).

**Table 2:** Significant differences in GMV in pairwise comparisons.

**Contrast**	**Hemisphere**	**Clusters**	**Peak (MNI)**			**Number of Voxels**	***T*-value**
			**X**	**Y**	**Z**		
**vSZ** **>** **nvSZ^a^**							
	L	Inferior parietal lobe	−51	−39	42	16	3.32
**vSZ** **<** **HC^b^**							
	R	Superior temporal gyrus	55	3	−13	2,854	−6.28
		Temporal Pole: superior temporal gyrus					
		Temporal Pole: middle temporal gyrus					
		Insula					
		Inferior frontal gyrus, orbital					
	R	Superior temporal gyrus	57	−22	4	102	−5.43
	L	Fusiform gyrus	−34	−13	−31	237	−5.60
	L	Inferior frontal gyrus, orbital	−48	19	−7	48	−5.16
	R	Inferior frontal gyrus, opercular	47	10	3	28	−5.12
**nvSZ < HC^b^**							
	L	Inferior parietal lobe	−58	−39	42	469	−5.63
	L	Parahippocampal	−18	−20	−22	192	−5.14
	R	Temporal Pole: superior temporal gyrus	49	9	−20	164	−5.46
	R	Inferior frontal gyrus, orbital	47	20	−11	333	−4.87
	L/R	Rectus, including bilateral medial orbital frontal gyrus	−1	40	−14	100	−5.11

*L, Left; R, Right; vSZ, violent offenders with schizophrenia; nvSZ, non-violent patients with schizophrenia*.

a *P_(voxel)_ < 0.001, uncorrected*.

b *P_(cluster)_ < 0.05, FWE corrected (family-wise error-corrected)*.

### Correlation Analyses

**Figure 4:**
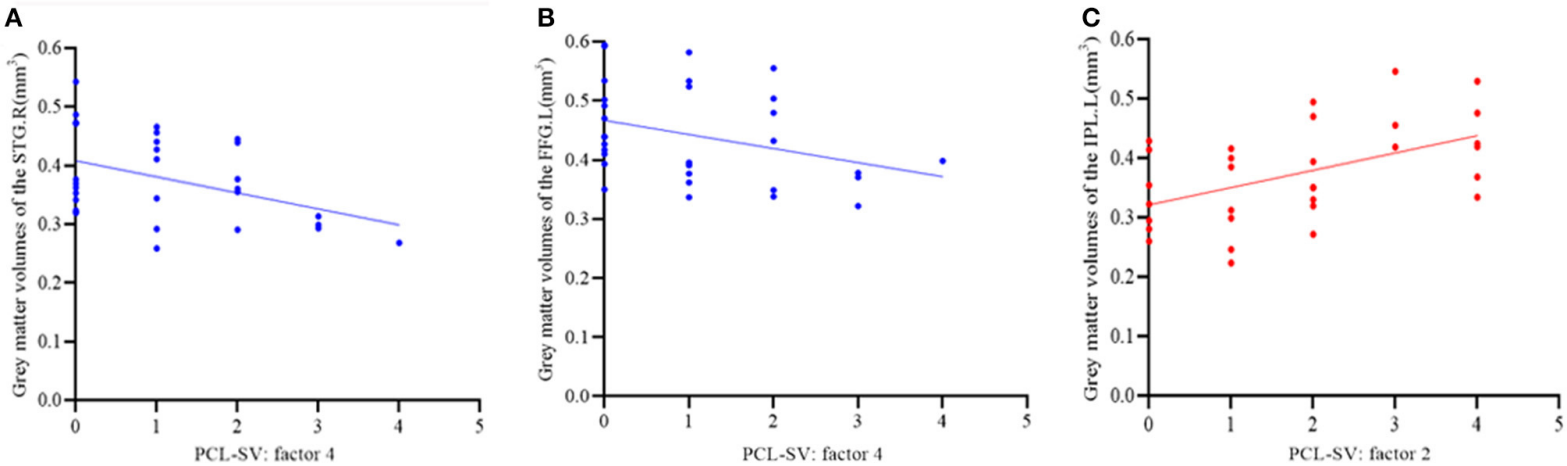
**(A,B)** Spearman correlation analysis of the gray matter volumes of the right superior temporal gyrus **(A)** and left fusiform gyrus **(B)** and antisocial trait of psychopathy in the vSZ group. **(C)** Spearman correlation analysis of the gray matter volumes of the left IPL and unemotional trait of psychopathy in the vSZ group.

In the vSZ group, significant negative correlations were found between the score of PCL:SV factor 4 (antisocial trait of psychopathy) and the GMVs in the right STG (*r* = −0.398, *p* = 0.027) and the left FFG (*r* = −0.357, *p* = 0.048) (Figures 4A,B); a negative correlation trend was also found between the score of PCL:SV factor 4 and the GMV in the right IFGoper (*r* = −0.351, *p* = 0.053). In contrary, there was a significant positive correlation between the score of the PCL:SV factor 2 (unemotional trait of psychopathy) and the GMV in the left IPL in the vSZ group (*r* = 0.506, *p* = 0.004) (Figure 4C). In the nvSZ group, no significant correlation was found between the total score as well as the score of each factor of the PCL-SV scale and the GMV in regions associated with violence. Regarding the clinical symptoms, we have found no significant correlation between the total score of the BPRS scale and imaging indicators in either SZ group.

## Discussion

The present study found a more pronounced decrease of GMV in vSZ, which involved the right STG extending to the TP and INS, as well as the left FFG and bilateral IFG, as compared to HCs; while decreased GMV in nvSZ was specifically located in the left IPL, PHG, and bilateral mOFG compared to HCs. Furthermore, the vSZ group showed a larger GMV in the left IPL than the nvSZ group. These results suggested that structural deficits in the frontotemporal lobe, particularly the temporal lobe, might be predispositions to violent behaviors in patients with SZ. The significant correlations between psychopathic traits and temporal structural deficits in the vSZ group indicate that the neurobiological underpinnings of violence in patients with SZ might be attributed to high levels of psychopathic traits and represent more of the characteristics of violence per se. The study provided the advantage of using the whole-brain approach to detect any regions associated with violence, as specific isolated regions (ROI-based analysis) were insufficient to reveal the mechanisms of complex phenotypes, especially violence (43). Furthermore, to improve the homogeneity of data and reduce the impact of confounders on neurobiological variability in relation to violence, all the patients were free of substance abuse and were comparable for clinical symptoms and medications.

Extensive research has elucidated the involvement of the temporal gyrus in violence, not only in patients with SZ (10, 44), but also in non-psychotic criminals (45, 46). For instance, Kuroki et al. (11) found great losses in the volume of the STG and TP, Liu et al. (47) also found significantly reduced GMVs in widespread frontal, temporal, and limbic regions in vSZ compared to HCs. A recent work demonstrated that violence in male violent patients with SZ is related to reduced cortical thickness in the temporal region and FFG (48). Our findings were consistent with these studies and support that violence in SZ is associated with morphological deficits in the right STG and its surrounding areas, which are involved in the processing of emotion-related information. Similar to these findings in vSZ, abnormal GMV was also prominent in violent offenders with psychopathy from forensic facilities (46, 49, 50). These results suggested a unique role of the STG in predisposing to violence in SZ by interfering with visual information processing and higher-order cognition related to emotional processing (51). Intriguingly, correlation analysis in this study further revealed a negative correlation between psychopathic traits (reflected by the antisocial score) and structural damage in the STG in the vSZ group, suggesting that abnormal GMV of the STG might predispose to violent behavior in patients with SZ, which might be fostered by the antisocial trait of psychopathy. By investigating the effect of psychopathy on the brain structure and function in vSZ, the present study has provided primary evidence that abnormal GMV in the STG might contribute to interpersonal violence in SZ.

In addition to the STG and TP, we also found significantly decreased GMV in the FFG in vSZ rather than nvSZ. This result is accord with other studies, which supported the malfunction of the FFG in the etiology of violence in patients with SZ (8, 17, 47). For instance, Liu (47) and Del Bene (17) found reduced GMV in the right FFG in vSZ compared to HCs, as well as reduced cortical thickness of this region compared to those with nvSZ. However, no correlation was reported between the aberrance in the FFG and any clinical characteristics. It is believed that the FFG plays an important role in face perception and visual processing, especially when viewing negative faces (52). Evidence on the neurodevelopmental abnormality of the FFG in patients with SZ (53, 54) has indicated that impairments in face recognition might be a prevalent deficit in SZ. However, the present study found a volumetric reduction in the FFG in the vSZ group only, and bridged the relationship between psychopathic traits (reflected by the antisocial score) and volumetric reduction in the FFG in the vSZ group, suggesting that a pronounced GMV reduction in the FFG might predispose to a persistent risk for violence in SZ due to deficits in emotion perception, especially for negative faces. Some functional imaging studies in individuals with psychopathy showed reduced FFG activation, which related to face processing (55, 56), supporting the association between impairments of the FFG-related face processing and violence in SZ.

In addition to the right STG and FFG, the right INS, as a hub of the emotional salience network (57), might be involved in violent behavior in patients with SZ (11) through socio-emotional information processing and experiencing other's pains and inner states (58). Furthermore, we have consistently found reduced GMV in the OFG in both patient groups. However, significantly reduced GMV in the IFG, which consists of the bilateral orbital and right opercular frontal gyri, has been found specific to violent patients. As part of the ventral prefrontal gyrus, the IFG is explicitly engaged in salient information integration and emotional regulation (59), in addition to inhibitory control (60). Previous studies have demonstrated that structural alterations of the IFG were related to aggressive behaviors in patients with SZ (61, 62). Therefore, our results further highlighted the association between the IFG and persistent violence in patients with SZ, especially a potential correlation of the IFGoper with psychopathic traits.

It is noteworthy that, on the one hand, a significant GMV reduction in the left IPL was observed in the nvSZ group only, indicating that the morphological deficits in the left IPL might be specific to the disease itself (63); on the other hand, the significant correlation between GMV in the left IPL and psychopathic traits in the present study might be explained by a more mild impairment in cognition in the vSZ group, as compared to the nvSZ group. For instance, Abu-akel et al. found that violent patients had more difficulties than non-violent patients in tasks involving empathic inference and better abilities in inferring cognitive mental states in others (64). Given the sparse findings of structural abnormality in the IPL associated with violence in SZ in prior studies, further studies are needed to determine whether the differences in the IPL between the patient groups are resulted from the impairment in cognition.

Given that GMV is the product of both cortical thickness and surface area (65, 66), it was shown that reduced cortical volume was associated with less numbers of neuronal and glial cells as well as reduced SA, sulcal depth, and region-specific cortical folding complexity. Our findings suggest there would be neurodevelopmental deficits in the frontotemporal network in violent patients with SZ. Inversely, increased cortical volume in the IPL might be associated with more neuronal and glial cells as well as increased microstructure of the cortical volume.

### Limitations

Some limitations in this study should be mentioned. First, the data of this study were analyzed at the voxel level, without a specific or personalized map to measure the structural variation of subcortical regions such as the amygdala and hippocampus using volumetric method due to their morphological anomalies. However, it seems that there has been no better method to delineate these structural changes. Second, limited information was obtained about the patients' history of medication, which precluded us from estimating the influence of intervention. Third, due to the small forensic sample, making it difficult to determine whether the results would be the same for individuals who had a high tendency for violence but were not incarcerated. Finally, a criminal cohort without psychosis was not included to examine the relationship between the diagnosis and violence; furthermore, since our study has not included participants with ASPD, it was likely to exclude those with high scores on PCL:SV factor 4, and this could have an effect on our results. Currently, we are collecting more data to expand the dataset for further analyses. In view of feasibility, no objective physical examination was completed for healthy controls other than verbal inquiries, thus, we might miss some existing physical problems in these participants.

## Conclusion

Our results extended the evidence that the structural abnormality of the frontotemporal network, especially the temporal lobe, which was involved in the regulation of emotion recognition, response and decision-making, was related to greater risk for violence in patients with SZ [for reviews (9, 44, 51)]. Our findings also provided knowledge about the association between psychopathic personality traits and the STG and FFG in patients with SZ who had violent behavior, suggesting that psychopathic traits, as a risk factor of violence, might be a predisposition to violent behaviors in patients with SZ through the frontotemporal network. In future studies, we need to take a closer look at psychopathic traits for better understanding of the mechanism of interpersonal violence in patients with SZ and to explore whether the imaging findings from this study can serve as a biomarker to predict future violent behaviors and community living.

## Data Availability Statement

The raw data supporting the conclusions of this article will be made available by the authors, without undue reservation.

## Ethics Statement

The studies involving human participants were reviewed and approved by Second Xiangya Hospital, Central South University. The patients/participants provided their written informed consent to participate in this study.

## Author Contributions

NG and JZ designed the study. NG analyzed the data and drafted the manuscript. XW and JZ revised the manuscript. NG, SZ, JL, XL, HG, and QS collected the clinical and MRI data. All authors contributed to and have approved the final manuscript.

## Funding

This study was supported by the National Natural Science Foundation of China (ID: 82171509 to XW, 82071543 to JZ), the Hunan Province Innovation Province Construction Project (Grant Number: 2019SK2334 to XW and JZ), the Natural Science Foundation of Hunan Province (2019JJ40424 to JZ), and the Health Committee of Hunan (202103091470 to JZ).

## Conflict of Interest

The authors declare that the research was conducted in the absence of any commercial or financial relationships that could be construed as a potential conflict of interest.

## Publisher's Note

All claims expressed in this article are solely those of the authors and do not necessarily represent those of their affiliated organizations, or those of the publisher, the editors and the reviewers. Any product that may be evaluated in this article, or claim that may be made by its manufacturer, is not guaranteed or endorsed by the publisher.
