# Supplementary material for: Structural Deficits in the Frontotemporal Network Associated With Psychopathic Traits in Violent Offenders With Schizophrenia
Source: Front Psychiatry. 2022 Apr 12;13:846838. doi: 10.3389/fpsyt.2022.846838 (PMC9039223; doi:10.3389/fpsyt.2022.846838)
Supplement: Supplementary file 1 [file Data_Sheet_1.pdf]

Supplementary Materials for

Structural deficits in the frontotemporal network associated with psychopathic traits in violent offenders with schizophrenia

Ningzhi Gou1, Juntao Lu1, Simei Zhang2, Xiaoxi Liang1, Huijuan Guo1, Qiaoling Sun1, Jiansong Zhou1*, Xiaoping Wang1*

* Correspondence author: e-mail: xiaop6@csu.edu.cn

Table S1 Significant differences in GMV in pairwise comparisons

Contrast	Hemisphere	Clusters	Peak (MNI)			Number of Voxels	*T* value
			X	Y	Z		
vSZ > nvSZ [a]							
	L	Inferior parietal lobe	-51	-39	42	23	3.32
	R	Inferior/Middle temporal gyrus	52	-36	-16	30	3.69
vSZ＜HC [b]							
	R	Superior temporal gyrus	55	3	-13	2854	-6.28
		Temporal Pole: superior temporal gyrus					
		Temporal Pole: middle temporal gyrus					
		Insula					
		Inferior frontal gyrus, orbital					
	R	Superior temporal gyrus	57	-22	4	102	-5.43
	L	Fusiform gyrus	-34	-13	-31	237	-5.60
	L	Inferior frontal gyrus, orbital	-48	19	-5	48	-5.16
	R	Inferior frontal gyrus, opercular	47	12	6	29	-5.12
nvSZ＜HC [b]							
	L	Inferior parietal lobe	-58	-39	42	463	-5.63
	L	Parahippocampal	-18	-20	-22	192	-5.63
	R	Temporal Pole: superior temporal gyrus	49	9	-20	164	-5.46
	R	Inferior frontal gyrus, orbital	47	20	-11	333	-5.29
	L/R	Rectus, including bilateral medial orbital frontal gyrus	-1	40	-14	100	-5.11

L: Left; R: Right; vSZ: violent offenders with schizophrenia; nvSZ: non-violent patients with schizophrenia;
[a] $P_{(voxel)} < 0.001$, uncorrected; [b] $P_{(cluster)} < 0.05$, FWE corrected (family-wise error-corrected).

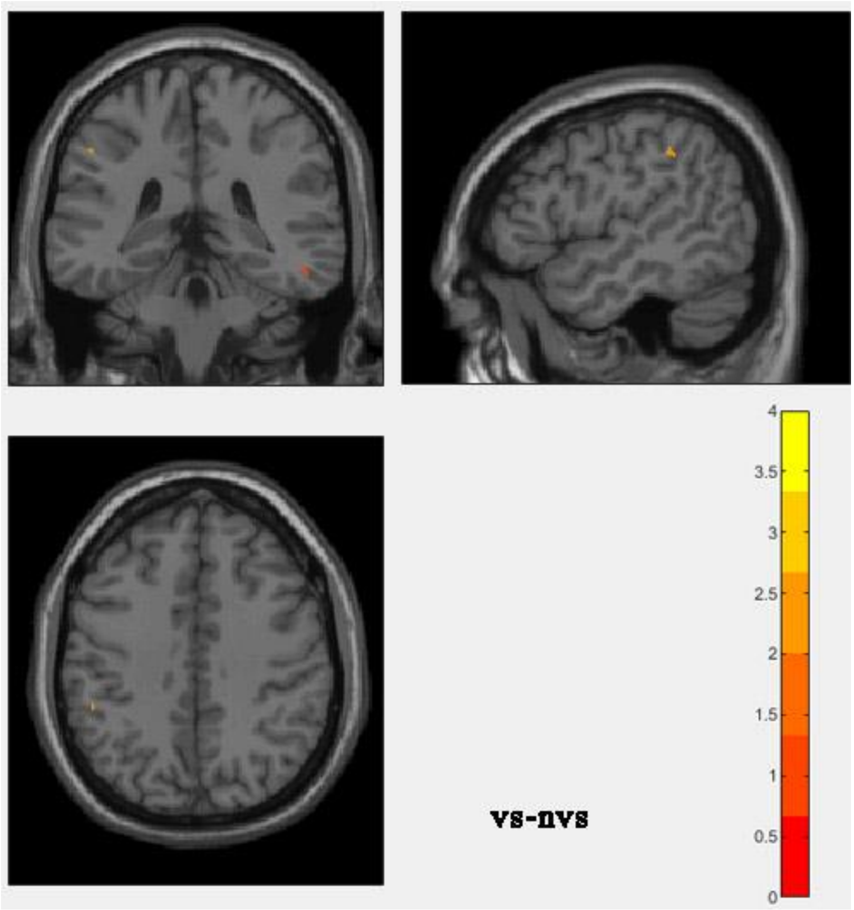

Figure S1. Larger gray matter volume in the vSZ group compared to the nvSZ group, with age, education, and TIV controlled. TIV: total intracranial volume.

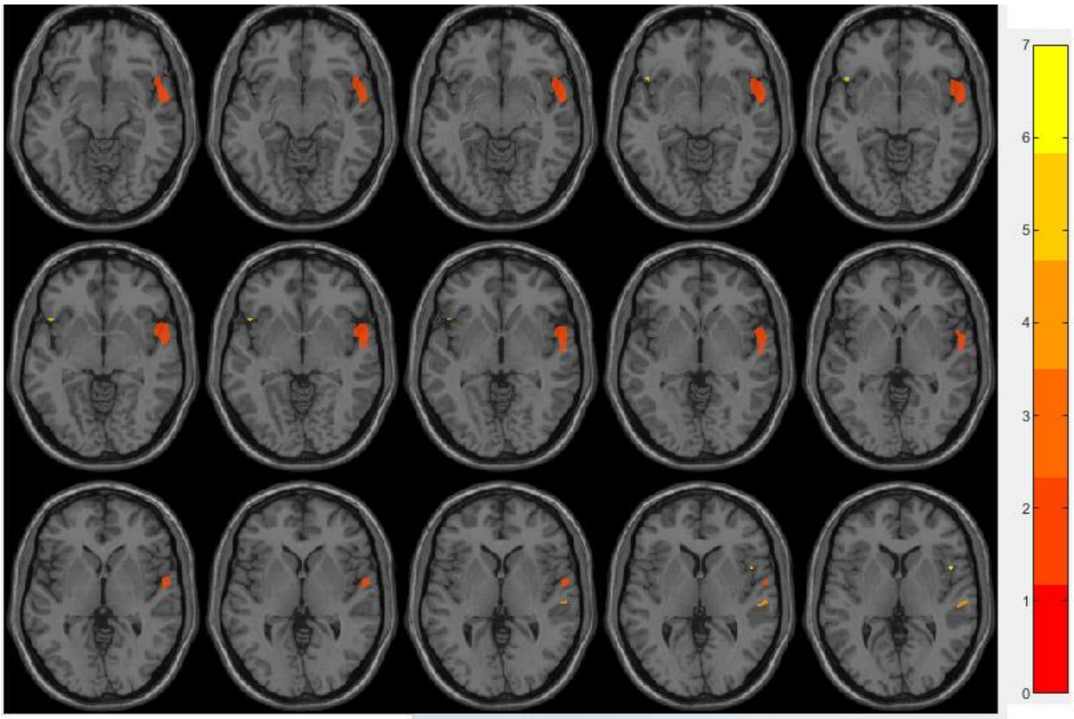

Figure S2. Reduced gray matter volume in the vSZ group compared to the HC group, with age, education, and TIV controlled. TIV: total intracranial volume.

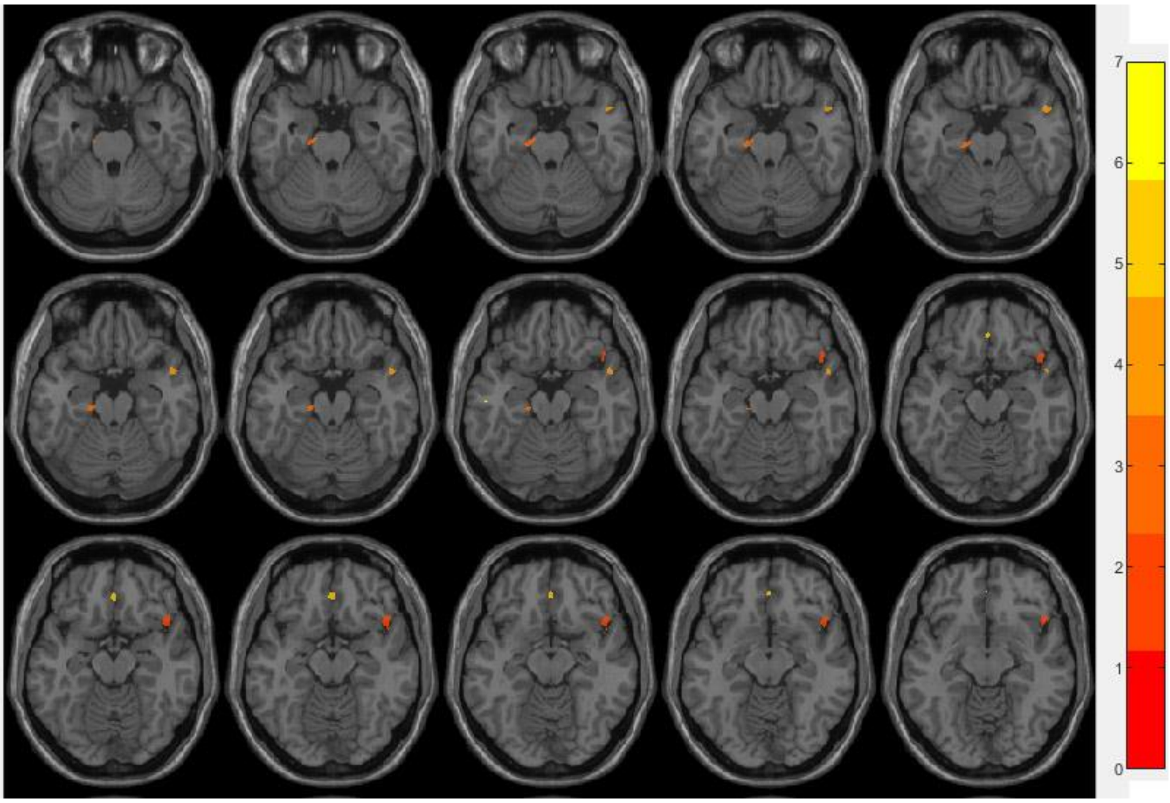

Figure S3. Reduced gray matter volume in the nvSZ group compared to the HC group, with age, education, and TIV controlled. TIV: total intracranial volume.
